# Peripheral and renal interstitial T-cell profiles associated with treatment response in lupus nephritis: a retrospective cohort study

**DOI:** 10.1093/ckj/sfag106

**Published:** 2026-03-26

**Authors:** Jingjing Wang, Yingxin Rong, Mengyue Zhu, Yuanmao Tu, Duqun Chen, Dandan Qiu, Feng Xu, Dandan Liang, Lin Chen, Haitao Zhang

**Affiliations:** National Clinical Research Center for Kidney Diseases, Jinling Hospital, Affiliated Hospital of Medical School, Nanjing University, Nanjing, China; National Clinical Research Center for Kidney Diseases, Jinling Hospital, Affiliated Hospital of Medical School, Nanjing University, Nanjing, China; Department of Nephrology, Northern Jiangsu People’s Hospital, Yangzhou, China; National Clinical Research Center for Kidney Diseases, Jinling Hospital, Affiliated Hospital of Medical School, Nanjing University, Nanjing, China; National Clinical Research Center for Kidney Diseases, Jinling Hospital, Affiliated Hospital of Medical School, Nanjing University, Nanjing, China; National Clinical Research Center for Kidney Diseases, Jinling Hospital, Affiliated Hospital of Medical School, Nanjing University, Nanjing, China; National Clinical Research Center for Kidney Diseases, Jinling Hospital, Affiliated Hospital of Medical School, Nanjing University, Nanjing, China; National Clinical Research Center for Kidney Diseases, Jinling Hospital, Affiliated Hospital of Medical School, Nanjing University, Nanjing, China; National Clinical Research Center for Kidney Diseases, Jinling Hospital, Affiliated Hospital of Medical School, Nanjing University, Nanjing, China; National Clinical Research Center for Kidney Diseases, Jinling Hospital, Affiliated Hospital of Medical School, Nanjing University, Nanjing, China

**Keywords:** lupus nephritis, mediation analysis, renal interstitial infiltration, T-cell profiles, treatment response

## Abstract

**Background:**

Lupus nephritis (LN) treatment response remains heterogeneous. We investigated associations between peripheral/renal T-cell profiles and treatment response, and explored renal T-cell infiltration as a mediator.

**Methods:**

This retrospective cohort study analyzed data from 424 LN patients. Peripheral CD4^+^/CD8^+^ T-cell counts were measured via flow cytometry, and renal interstitial infiltrations were assessed immunohistochemically. Associations with treatment response were evaluated using generalized linear and logistic regression models, adjusting for clinicopathological factors. Mediation analysis examined renal T-cell infiltration in connecting peripheral immunity and treatment outcomes.

**Results:**

The study cohort consisted of 424 patients with biopsy-confirmed LN, predominantly female (84.67%), with a mean age of 30.37 ± 11.18 years. Responders, comprising 68.4% of the cohort, exhibited significantly higher peripheral CD4^+^ T-cell counts (median 310 vs. 265 cells/μl, *P* = .002) and CD4/CD8 ratios (0.94 vs. 0.73, *P* < .01), with adjusted OR of 1.002 (95% CI 1.001–1.003) and 2.462 (95% CI 1.414–4.288), respectively. These associations remained significant after Bonferroni correction. Nonresponders showed increased renal interstitial CD8^+^ T-cell infiltration (148 vs. 80 cells/mm^2^, *P* < .001), while higher renal interstitial CD4/CD8 ratios predicted remission (OR = 8.312, 95% CI 2.593–26.645). The peripheral CD4/CD8 ratio provided incremental predictive value over standard clinical-pathological indices (AUC improvement: 0.711 vs. 0.678, *P* = .022). Mediation analysis revealed that the renal interstitial CD4/CD8 ratio mediated 11.97% of the total effect of the peripheral CD4/CD8 ratio on treatment response (indirect effect β = 0.011, *P* = .016).

**Conclusion:**

Peripheral and renal interstitial T-cell profiles, particularly CD4/CD8 ratios, are significantly associated with treatment response in LN. Renal interstitial T-cells partially mediate the impact of peripheral immune status on clinical outcomes.

## GRAPHICAL ABSTRACT

**GRAPHICAL ABSTRACT:**
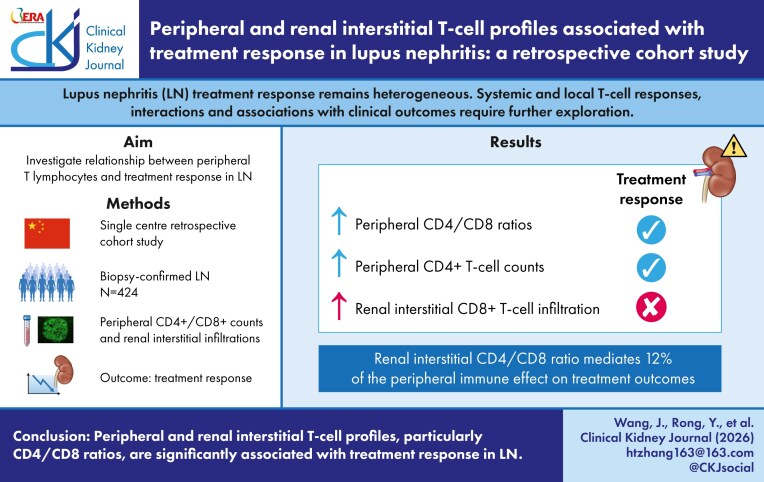

KEY LEARNING POINTS **What was known:**

- T-cell dysregulation in LN pathogenesis, especially CD4^+^/CD8^+^ imbalance, links to disease activity.
- Renal CD8^+^ T-cell infiltration dominates in LN and correlates with histological damage severity.
- Lack of biomarkers to predict treatment response in LN, where 30%–40% patients are refractory.

**This study adds:**

- Peripheral CD4/CD8 ratios and renal CD8 density are strongly associated with treatment response in lupus nephritis patients.
- Renal interstitial CD4/CD8 ratio mediates 11.97% of the peripheral immune effect on treatment outcomes.
- Combined peripheral and renal T-cell profiling offers superior prognostic stratification compared to individual assessments.

**Potential impact:**

- Enables risk stratification via accessible peripheral blood tests and renal biopsy quantification for personalized therapy.
- Identifies renal T-cell infiltration as a novel drug target for refractory LN patients.

## BACKGROUND

Lupus nephritis (LN), a severe kidney complication of systemic lupus erythematosus (SLE), results from immune complex deposits in the glomeruli, triggering inflammation and kidney damage [1]. Standard treatment involves glucocorticoids and immunosuppressants, with biological agents like belimumab and obinutuzumab being used more frequently [2–4]. Despite this, 30%–40% of patients remain refractory to treatment, highlighting the need for biomarkers to predict treatment outcomes [5–7].

T-cell dysregulation is central to LN pathogenesis, yet the clinical relevance of specific T-cell subsets remains ambiguous. Although T cells promote renal injury through direct cytotoxicity and B-cell help [8], their subset dynamics exhibit contradictory associations. Peripherally, reduced CD4^+^/CD8^+^ ratios correlate with higher disease activity [9], and CD8^+^ T-cell persistence predicts immunosuppressive resistance [10].

Furthermore, T cells are the predominant renal infiltrating cells in both humans and mouse lupus models [11]. Renally, CD8^+^ T cells dominate infiltrates across all LN classes and outnumber CD4^+^ cells in all renal compartments. Crucially, intrarenal CD8^+^ density directly associates with histologic severity, whereas CD4^+^ infiltration shows no such correlation [12]. Paradoxically, urinary CD4^+^/CD8^+^ ratios are elevated and linked to proteinuria [13].

There are still significant gaps in understanding how systemic and localized T-cell responses interact. Most studies have looked at peripheral lymphocytes or kidney infiltrates separately. The role of kidney T-cell infiltration in linking peripheral immunity to clinical outcomes hasn’t been fully explored. This study aims to investigate the connection between peripheral T lymphocytes and treatment response in LN, while also considering the mediating role of kidney T-cell infiltration.

## MATERIALS AND METHODS

### Study design and patient selection

**Figure 1:**
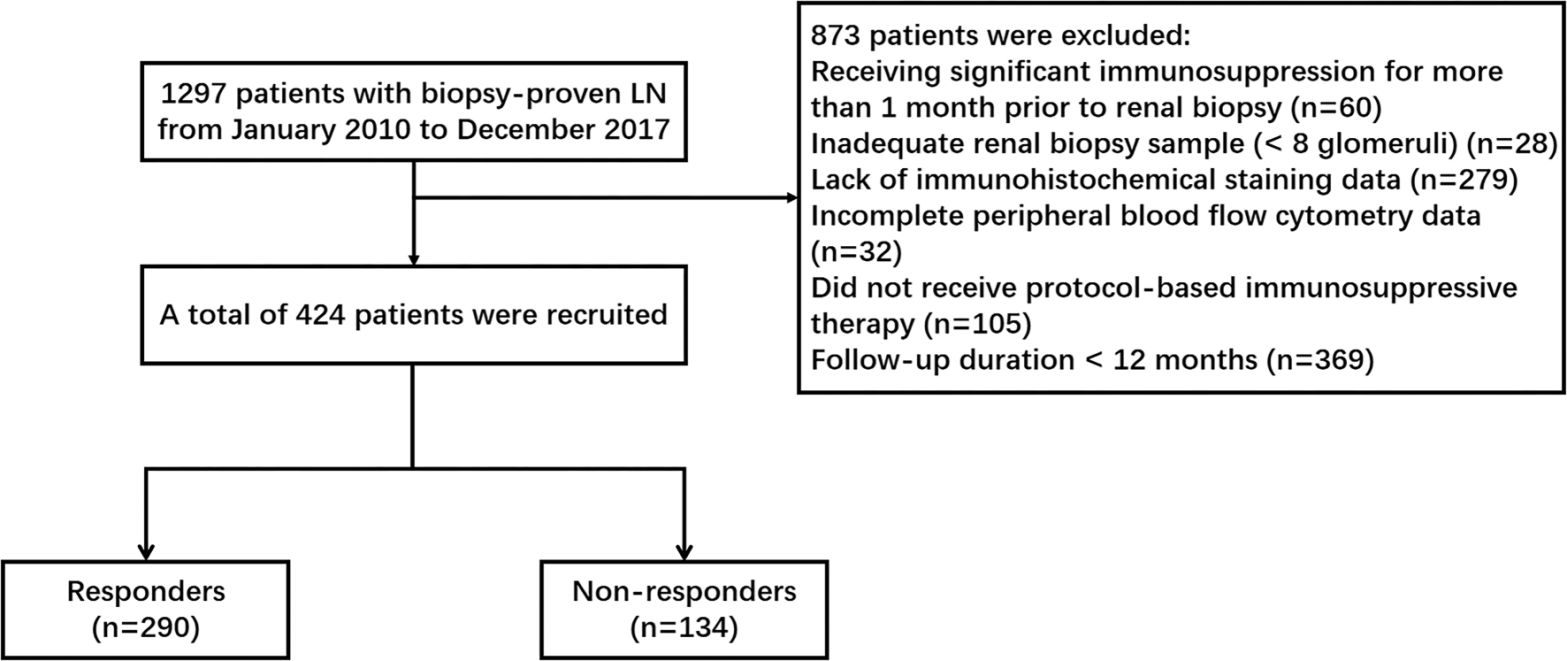
Flowchart of the study.

This single-center retrospective cohort included biopsy-proven LN patients treated at Nanjing Jinling Hospital (2010–2017). Inclusion criteria required patients to meet at least four of the criteria for SLE as defined by the 1997 revised classification of the American College of Rheumatology. Renal biopsy samples were evaluated by the classification criteria of the International Society of Nephrology/Renal Pathology Society (ISN/RPS). All participants were monitored for at least 12 months. Exclusion criteria included patients with incomplete flow cytometry or biopsy data. Many patients had a pre-existing SLE diagnosis, but we excluded any patient who had received significant immunosuppression (excluding a prednisone dose or equivalent not exceeding 0.5 mg·kg^−1^·d^−1^) for more than one month before the biopsy (Fig. 1). The study protocol was approved by the Institutional Review Board (Grant Number: 2024DZKY-046-01).

### Data collection and definition

Demographic, clinicopathological characteristics, treatment regimens, and patient outcomes were extracted from electronic medical records. Peripheral blood samples for flow cytometry were collected within 72 hours before the renal biopsy, before the initiation of any significant immunosuppression for the current LN flare (defined as a prednisone dose or equivalent not exceeding 0.5 mg·kg^−1^·d^−1^).

These samples were analyzed by flow cytometry using a Cytomics FC 500 instrument (Beckman Coulter) and the Beckman Coulter CD45/CD4/CD8/CD3 reagent kit (Cat No: 6607013), containing the following fluorochrome-conjugated antibodies: CD45-FITC (clone B3821F4A), CD3-PC5 (clone UCHT1), CD4-RD1 (clone SFCI12T4D11), and CD8-ECD (clone SFCI21Thy2D3). The procedure strictly adhered to the International Society for Advancement of Cytometry (ISAC) guidelines. Lymphocytes were gated based on forward and side scatter properties. CD4^+^ T-cells were defined as CD45^+^CD3^+^CD4^+^CD8^−^, and CD8^+^ T-cells were defined as CD45^+^CD3^+^CD8^+^CD4^−^. Absolute counts of CD3^+^CD4^+^ and CD3^+^CD8^+^ T cells were determined using Flow-Count fluorospheres (Beckman Coulter, Inc.) as per ISAC standards. The CD4/CD8 ratio was subsequently calculated by dividing the absolute count of CD4^+^ T cells by the absolute count of CD8^+^ T cells.

Induction therapy was tailored based on renal biopsy findings, with adjustments made by the treating physician according to prevailing guidelines. Dosing protocols for all regimens included (e.g. IV cyclophosphamide (CYC) 0.5–1.0 g/m^2^ monthly; oral mycophenolate mofetil (MMF) target dose 1–2 g/day; starting oral prednisone 0.5–0.6 mg/kg/day; CNI dosages adjusted based on blood concentration). Patients received pulse IV methylprednisolone (e.g. 500–1000 mg for 3 days) prior to oral steroids.

The treatment response was categorized into complete remission (CR), defined as quantitative urinary protein quantitation (UPRO) less than 0.4 g/24 hours and normal serum creatinine (SCr) levels, and partial remission (PR) characterized by a reduction in proteinuria of at least 50%, UPRO of less than 3.5 g/24 hours, serum albumin levels of at least 30 g/l, and either normal SCr levels or no more than 15% increase from baseline. Responders were identified as those achieving either complete or PR within 12 months of treatment initiation. Patients who did not achieve complete or PR after 12 months of immunosuppressive therapy were classified as nonresponders.

### Renal histopathology

Renal histopathology analysis was conducted on renal biopsy specimens obtained from percutaneous needle biopsy, which were routinely examined using light microscopy, immunofluorescence, and electron microscopy. The biopsies were independently reviewed by two nephropathologists. Pathological parameters, including activity indices (AIs) and chronicity indices (CIs), were assessed following established methodologies. The individual components of the NIH Activity and Chronicity indices were scored as previously described, and the detailed breakdown is provided in Supplementary Table S1. Kidney tubular lesions were scored semi-quantitatively as follows: 0 indicated no lesions present; 1 score indicated lesions present in 1%–24% of the sample; 2 scores indicated lesions present in 25%–49% of the sample; and 3 scores indicated lesions present in 50% or more of the sample.

Infiltrating lymphocytes in renal tissue were identified using immunohistochemical staining. Paraffin-embedded tissue sections, each measuring 2 µm in thickness, were incubated with primary antibodies, specifically rabbit anti-human CD3 (SP7; LEICA), rabbit anti-human CD4 (NCL-L-368; LEICA), and rabbit anti-human CD8 (NCL-L-295). Following this, the slides were incubated with a secondary antibody, and visualization was achieved using a LEICA System Kit with hematoxylin serving as the counterstain. The stained sections were subsequently scanned using a Digital Pathology Slide Scanner (Leica, Wetzlar, Germany). Positive-stained cells were automatically quantified in all nonglobally sclerotic glomeruli at 400× magnification using Aperio eSlide Manager (Leica). The counts were normalized for area, and the unit reported is cells/mm^2^.

### Statistical analysis

We performed a comparative analysis of the levels of each peripheral blood T lymphocyte subtype and renal interstitial T lymphocyte subtype infiltrate (CD4, CD8, and CD4/CD8 ratio) between responders and nonresponders using the Mann-Whitney test. To evaluate the relationship between T lymphocyte subtype levels and various pathological and clinical variables, Spearman correlation coefficients were calculated. Logistic regression analyses were utilized to investigate the association between tertiles of peripheral blood and renal interstitial T lymphocyte subtypes and treatment response, including adjusted and unadjusted gender, age, SLEDAI, hypertension, LN duration, estimated glomerular filtration rate (eGFR), UPRO, Anti-dsDNA, C3, ISN/RPS classification, AI, CI, acute renal tubular injury, interstitial fibrosis and tubular atrophy (IFTA), and therapy class. Additionally, generalized linear models (including linear regression for continuous variables and logistic regression for binary outcomes) were conducted to assess the associations between the CD4/CD8 ratios in peripheral blood and renal interstitial inflammatory cell profiles, as well as remission within 12 months. These statistical methods were also employed in subgroup analyses to explore potential differences among specific populations, including those defined by gender, age, SLEDAI, LN duration, hemoglobin levels, eGFR, UPRO, ISN/RPS classification, AI, and CI.

To evaluate the incremental predictive value of the peripheral CD4/CD8 ratio over established prognostic factors, we compared the performance of a baseline clinical-pathological model (including SLEDAI, eGFR, UPRO, AI, and CI) against an enhanced model that added the peripheral CD4/CD8 ratio. Model discrimination was compared using DeLong’s test for the area under the receiver operating characteristic curve (AUC). Net reclassification improvement and integrated discrimination improvement were also calculated.

The “mediation” package in R version 4.2.3 was employed to conduct a mediation analysis aimed at evaluating the mediating effects of the renal interstitial infiltration CD4/CD8 ratios on the relationship between the CD4/CD8 ratios in peripheral blood and remission within 12 months. This analysis was adjusted for variables including gender, age, SLEDAI, duration of LN, hemoglobin levels, eGFR, UPRO, ISN/RPS classification, activity index (AI), chronicity index (CI), acute renal tubular injury, IFTA, and treatment class.

To assess the association between baseline T-cell profiles and long-term renal prognosis, we performed survival analysis using the composite endpoint of doubling of SCr from baseline or progression to end-stage kidney disease (ESKD). Time to event was calculated from the date of biopsy. Patients were stratified by the median peripheral CD4/CD8 ratio into high and low groups. Kaplan-Meier curves were plotted and compared using the log-rank test. Univariable and multivariable Cox proportional hazards models were used to estimate hazard ratios (HRs) with 95% confidence intervals (CIs). Multivariable models were adjusted as specified in the results.

Bonferroni correction was applied for multiple comparisons to control the family-wise error rate. Statistical significance was defined as a two-tailed *P* < .05, and findings that remained significant after Bonferroni correction are explicitly indicated. All analyses were performed in R v4.2.3.

## RESULTS

### Baseline clinical and pathological characteristics

The cohort comprised 424 biopsy-proven LN patients (84.67% female; mean age 30.37 ± 11.18 years), with detailed demographics and clinicopathological features summarized in Tables 1 and 2. Proliferative LN (ISN/RPS class III/IV ± V) accounted for 87.26% of cases (Table 3). Among the included patients, 127 had received no prior treatment, and 297 patients received treatment with low to moderate doses of prednisone. Only four patients underwent a repeated kidney biopsy.

**Table 1:** Baseline demographic and clinical characteristics of lupus nephritis patients stratified by response to immunosuppression.

Characteristics	Total *N* = 424	Nonresponders *n* = 134	Responders *n* = 290	*P*-value
**Demographics**				
**Age (years)**	30.37 ± 11.18	29.64 ± 10.99	30.70 ± 11.27	.364
**Male gender, *n* (%)**	65 (15.33%)	36 (26.87%)	29 (10.00%)	**<.001**
**Disease history**				
**SLE duration (months)**	6.00 (1.00–43.50)	12.00 (1.00–48.00)	6.00 (1.00–36.00)	**.012**
**LN duration (months)**	2.00 (0.67–24.00)	4.00 (1.00–36.00)	1.25 (0.67–12.00)	**.022**
**Immunosuppressive therapy-naive, *n* (%)**	127(29.95%)	27(20.15%)	100(34.48%)	**.003**
**Disease activity**				
**SLEDAI (2K) (scores)**	14.36 ± 5.29	13.65 ± 4.90	14.69 ± 5.44	.061
**Co-morbidities, *n* (%)**				
**Hypertension**	144 (33.96%)	58 (43.28%)	86 (29.66%)	**.006**
**Diabetes**	40 (9.43%)	14 (10.45%)	26 (8.97%)	.627
**Extrarenal organ-system involvement, *n* (%)**				
**Cutaneous involvement**	225 (53.07%)	68 (50.75%)	157 (54.14%)	.55
**Haematologic disorder**	240 (56.60%)	83 (61.94%)	157 (54.14%)	.132
**Arthritis**	196 (46.23%)	65 (48.51%)	131 (45.17%)	.522
**Serositis**	62 (14.62%)	27 (20.15%)	35 (12.07%)	**.029**
**Neurological disorder**	18 (4.25%)	6 (4.48%)	12 (4.14%)	.872
**Laboratory parameters**				
**Hb (g/l)**	101.92 ± 19.98	99.63 ± 22.52	102.99 ± 18.64	.108
**ALB(g/l)**	28.60 ± 5.88	27.98 ± 5.89	28.88 ± 5.86	.146
**SCr (mg/dl)**	0.84 (0.63–1.31)	1.13 (0.67–2.21)	0.78 (0.62–1.08)	**<.001**
**UA (μmol/l)**	422.15 ± 138.93	460.26 ± 162.43	404.54 ± 122.98	**<.001**
**eGFR (ml/min per 1.73 m^2^)**	88.38 ± 38.98	74.54 ± 44.46	94.78 ± 34.41	**<.001**
**UPRO (g/24 h)**	3.10 (1.72–5.80)	3.08 (1.82–6.54)	3.13 (1.69–5.47)	.528
**Urinary red blood cell count (µl)**	63.50 (10.00–256.25)	53.20 (10.00–168.25)	67.00 (10.50–290.00)	.234
**Positive ANA, *n* (%)**	408 (96.23%)	125 (93.28%)	283 (97.59%)	**.031**
**Positive anti-dsDNA, *n* (%)**	242 (57.08%)	69 (51.49%)	173 (59.66%)	.114
**C3 (g/l)**	0.42 (0.30–0.57)	0.43 (0.31–0.60)	0.40 (0.30–0.56)	.073

SLE, systemic lupus erythematosus; LN, lupus nephritis; SLEDAI, systemic lupus erythematosus disease activity; Hb, hemoglobin; ALB, albumin; SCr, serum creatinine; eGFR, the estimated glomerular filtration rate; UA, blood uric acid; UPRO, urinary protein quantitation; ANA, anti-nuclear; anti-dsDNA, anti-double-stranded DNA antibodies; C3, component 3.

Bold values indicate statistically significant differences between responders and non-responders (P < 0.05). SLE, systemic lupus erythematosus; LN, lupus nephritis; SLEDAI, Systemic Lupus Erythematosus Disease Activity Index; Hb, hemoglobin; ALB, albumin; SCr, serum creatinine; eGFR, estimated glomerular filtration rate; UA, uric acid; UPRO, urinary protein quantitation; ANA, antinuclear antibody; anti-dsDNA, anti-double-stranded DNA antibody; C3, complement 3.

**Table 2:** Baseline treatment regimens and T-cell profiles stratified by response to immunosuppression.

Characteristics	Total *N* = 424	Nonresponders *n* = 134	Responders *n* = 290	*P*-value
**Peripheral T-cell profiles**				
**Peripheral CD3^+^ cell counts (μl)**	797.50 (550.75–1080.50)	754.50 (529.25–1052.25)	803.50 (567.25–1086.75)	.333
**Peripheral CD4^+^ cell counts (μl)**	299.50 (214.00–452.00)	265.00 (192.50–397.50)	310.00 (222.25–470.50)	**.002**
**Peripheral CD8^+^ cell counts (μl)**	407.50 (266.00–595.50)	436.50 (272.50–616.25)	398.50 (265.00–584.50)	.519
**Peripheral CD4/CD8 ratios**	0.78 (0.49–1.09)	0.64 (0.39–0.93)	0.84 (0.55–1.12)	**<.001**
**Treatment regimens, *n* (%)**				
**Pred + MMF + CNIs**	123 (29.01%)	39 (29.10%)	84 (28.97%)	.77
**Pred + CYC**	110 (25.94%)	39 (29.10%)	71 (24.48%)	.313
**Pred + MMF**	74 (17.45%)	20 (14.93%)	54 (18.62%)	.351
**Pred + CNIs**	67 (15.80%)	22 (16.42%)	45 (15.52%)	.83
**Others**	50 (11.79%)	14 (10.45%)	36 (12.41%)	.559

Pred, prednisone; MMF, mycophenolate mofetil; CNIs, calcineurin inhibitors; CYC, cyclophosphamide.

Bold values indicate statistically significant differences between responders and non-responders (P < 0.05). Pred, prednisone; MMF, mycophenolate mofetil; CNIs, calcineurin inhibitors; CYC, cyclophosphamide.

**Table 3:** Pathological characteristics of lupus nephritis patients stratified by response to immunosuppression.

Characteristics	Total *N* = 424	Nonresponders *n* = 134	Responders *n* = 290	*P*-value
**ISN/RPS classification, *n* (%)**				.916
**Class II**	9 (2.12%)	2 (1.49%)	7 (2.41%)	
**Class III**	30 (7.08%)	9 (6.72%)	21 (7.24%)	
**Class III + V**	44 (10.38%)	15 (11.19%)	29 (10.00%)	
**Class IV**	172 (40.57%)	52 (38.81%)	120 (41.38%)	
**Class IV + V**	124 (29.25%)	39 (29.10%)	85 (29.31%)	
**Class V**	45 (10.61%)	17 (12.69%)	28 (9.66%)	
**Activity index**	6.50 (4.00–9.00)	6.00 (4.00–9.00)	7.00 (4.00–9.00)	.935
**Chronicity index**	2.00 (1.00–3.00)	2.00 (1.00–4.00)	1.00 (0.00–3.00)	**<.001**
**Glomerulosclerosis (%)**	1.75 (0.00–8.30)	4.05 (0.00–12.32)	0.00 (0.00–5.00)	**<.001**
**Focal segmental glomerulosclerosis (%)**	0.00 (0.00–5.90)	0.00 (0.00–9.30)	0.00 (0.00–4.20)	**<.001**
**Crescents (%)**	8.75 (0.00–21.75)	11.26 (2.73–28.38)	7.30 (0.00–18.35)	**.003**
**CD4^+^cells/mm²**	92.00 (48.00–168.00)	136.00 (72.00–199.00)	80.00 (44.00–144.00)	**<.001**
**CD8^+^cells/mm²**	96.00 (51.00–168.00)	148.00 (72.00–207.00)	80.00 (40.00–144.00)	**<.001**
**Renal interstitial infiltration CD4/CD8 ratios**	1.00 ± 0.22	0.95 ± 0.23	1.02 ± 0.21	**.004**
**Acute renal tubular injury, *n* (%)**				**.015**
**0**	167 (39.39%)	45 (33.58%)	122 (42.07%)	
**1**	188 (44.34%)	57 (42.54%)	131 (45.17%)	
**2**	48 (11.32%)	20 (14.93%)	28 (9.66%)	
**3**	21 (4.95%)	12 (8.96%)	9 (3.10%)	
**IFTA, *n* (%)**				**<.001**
**0**	264 (62.26%)	65 (48.51%)	199 (68.62%)	
**1**	141 (33.25%)	55 (41.04%)	86 (29.66%)	
**2**	13 (3.07%)	8 (5.97%)	5 (1.72%)	
**3**	6 (1.42%)	6 (4.48%)	0 (0.00%)	

IFTA, interstitial fibrosis and tubular atrophy.

Bold values indicate statistically significant differences between responders and non-responders (P < 0.05). ISN/RPS, International Society of Nephrology/Renal Pathology Society; IFTA, interstitial fibrosis and tubular atrophy.

All patients completed the primary 12-month follow-up for assessment of treatment response. Treatment regimens were tailored based on the histopathological findings from renal biopsies. All patients received corticosteroid therapy, barring contraindications. Furthermore, 123 patients (29.01%) underwent multitarget therapy, 110 (25.94%) received CYC, 74 (17.45%) were treated with MMF, and 67 (15.80%) were administered calcineurin inhibitors, of which 60 were treated with tacrolimus and 7 with cyclosporine. The “Other” treatments category (*n* = 50, 11.79%) included: Triptergium wilfordii (*n* = 12, 2.83%), rituximab (*n* = 3, 0.71%), azathioprine (*n* = 10, 2.36%), autologous hematopoietic stem cell transplantation (*n* = 5, 1.18%), and corticosteroids alone (*n* = 20, 4.72%) (Table 2).

### Peripheral and renal T-cell profiles associated with treatment remission

The baseline characteristics of patients at the time of kidney biopsy were stratified based on their immunosuppressive response within 12 months, with renal remission observed in 290 patients (68.40%), as detailed in Tables 1 and 2. Significant differences were identified between the responder and nonresponder groups. Responders had a shorter duration of SLE (*P* = .012) and LN (*P* = .022), a lower prevalence of hypertension (*P* = .06), and lower SCr levels (*P* < .01) and decreased uric acid levels (*P* < .01). Pathologically, responders exhibited a lower CI (*P* < .01), a lower incidence of global sclerosis (*P* < .01), focal segmental glomerulosclerosis (FSGS) (*P* < .01), and crescents (*P* = .003). Additionally, they demonstrated a lower degree of acute renal tubular injury (*P* = .015) and IFTA (*P* < .01). (Table 3).

We performed a new analysis comparing Nonremission vs. PR vs. CR using multinomial logistic regression. A higher CD4/CD8 ratio was significantly associated with both partial remission (OR = 2.74, 95% CI: 1.47–5.12, *P* = .002) and CR (OR = 2.37, 95% CI: 1.32–4.26, *P* = .004) when compared to nonremission. These associations remained statistically significant after applying the Bonferroni correction for multiple comparisons (Adjusted *P*-value < .025) (Supplementary Table S2).

The analysis of T lymphocyte profiles in patients with LN showed distinct patterns between responders and nonresponders. Responders had higher circulating CD4 counts, higher CD4/CD8 ratios in the blood, and decreased interstitial CD4 and CD8 densities but increased CD4/CD8 ratios in the tubulointerstitium. Higher peripheral CD4^+^ T-cell counts were associated with lower SLEDAI, lower anti-dsDNA, and higher C3, while peripheral CD8^+^ T-cell counts also correlated with lower SLEDAI and higher C3 in correlation analysis. These profiles correlated with clinical and pathological parameters, including gender, age, SLEDAI, LN duration, UPRO, eGFR, Anti-dsDNA, C3, AI, CI, acute renal injury, and IFTA, with a maximum correlation coefficient of −0.4327. Circulating CD4 and CD8 counts were positively correlated with eGFR and C3 and negatively correlated with SLEDAI and Anti-dsDNA, while the CD4/CD8 ratios were negatively correlated with the CI. Lymphocyte infiltration, especially CD4 and CD8 cells, was correlated positively with UPRO, C3, AI, CI, acute renal tubular injury, and IFTA, while being negatively associated with eGFR. The renal interstitial CD4/CD8 ratio demonstrated a negative correlation with SLEDAI and anti-dsDNA levels; however, these correlations did not retain statistical significance following Bonferroni correction (Table 4).

**Table 4:** Correlation between peripheral/renal interstitial T lymphocyte subsets and clinicopathological variables in LN.

		Gender	Age	SLEDAI	LN duration	UPRO	eGFR	Anti-dsDNA	C3	Activity index	Chronicity index	Acute renal tubular injury	IFTA
Peripheral CD4^+^ T lymphocyte	Rho	−0.0600	0.0571	−0.2204	0.0238	−0.0235	0.1905	−0.1164	0.1131	−0.1264	−0.0560	−0.0467	−0.0633
	*P*-value	0.2179	0.2403	<0.0001	0.6251	0.6293	0.0001	0.0165	0.0198	0.0091	0.2499	0.3369	0.1935
	Adj. *P*-value	1.000	1.000	0.0072	1.000	1.000	0.0072	1.000	1.000	0.6552	1.000	1.000	1.000
Peripheral CD8^+^ T lymphocyte	Rho	−0.0501	−0.1068	−0.1907	0.1150	0.0222	0.1907	−0.1246	0.1456	−0.0669	0.0283	−0.0187	−0.0222
	*P*-value	0.3032	0.0279	0.0001	0.0178	0.6491	0.0001	0.0102	0.0027	0.1694	0.5605	0.7003	0.6479
	Adj. *P*-value	1.000	1.000	0.0072	1.000	1.000	0.0072	0.7344	0.1944	1.000	1.000	1.000	1.000
Peripheral CD4/CD8 ratio	Rho	−0.0228	0.1541	−0.0843	−0.0678	−0.0505	0.0427	−0.0026	−0.0148	−0.0509	−0.1172	−0.0354	−0.0685
	*P*-value	0.6399	0.0015	0.0831	0.1636	0.2991	0.3806	0.957	0.7618	0.296	0.0158	0.4675	0.1594
	Adj. *P*-value	1.000	0.1080	1.000	1.000	1.000	1.000	1.000	1.000	1.000	1.000	1.000	1.000
Renal interstitial CD4^+^ T lymphocyte	rho	0.1189	0.0876	0.0406	0.1362	0.1814	−0.4327	−0.0848	0.0966	0.1784	0.3286	0.2698	0.3266
	*P*-value	0.0143	0.0715	0.4041	0.005	0.0002	<0.0001	0.0813	0.0468	0.0002	<0.0001	<0.0001	<0.0001
	Adj. *P*-value	1.000	1.000	1.000	0.3600	0.0144	<0.0001	1.000	1.000	0.0144	<0.0001	<0.0001	<0.0001
Renal interstitial CD8^+^ T lymphocyte	rho	0.0980	0.0732	0.0707	0.1423	0.1822	−0.4208	−0.0598	0.0979	0.1557	0.3325	0.2576	0.3300
	*P*-value	0.0438	0.1323	0.1462	0.0033	0.0002	<0.0001	0.2193	0.0439	0.0013	<0.0001	<0.0001	<0.0001
	Adj. *P*-value	1.000	1.000	1.000	0.2376	0.0144	<0.0001	1.000	1.000	0.0936	<0.0001	<0.0001	<0.0001
Renal interstitial CD4/CD8 ratios	rho	0.0641	0.0586	−0.1375	−0.0203	−0.0089	0.0256	−0.1031	0.0201	0.0292	−0.0097	−0.0136	−0.0110
	*P*-value	0.1875	0.2288	0.0046	0.6764	0.8544	0.5994	0.0338	0.6794	0.5488	0.8427	0.7798	0.8216
	Adj. *P*-value	1.000	1.000	0.3312	1.000	1.000	1.000	1.000	1.000	1.000	1.000	1.000	1.000

SLEDAI, Systemic Lupus Erythematosus Disease Activity; UPRO, urinary protein quantitation; eGFR, the estimated glomerular filtration rate; C3, component 3; IFTA, Interstitial fibrosis and tubular atrophy.

Indicates that the correlation remains statistically significant after Bonferroni correction (Adjusted *P*-value < .000694).

To explore the relationship between circulating and renal T cells, we performed a correlation analysis. A weak but statistically significant inverse correlation was observed between peripheral and renal interstitial CD4⁺ T-cell densities (Spearman’s ρ = −0.120, *P* = .014). No such correlation was found for CD8⁺ T cells (ρ = −0.041, *P* = .399). The complete results of this analysis are provided in Supplementary Table S3.

### Associations of peripheral and renal interstitial T lymphocyte profiles with renal outcomes

**Figure 2:**
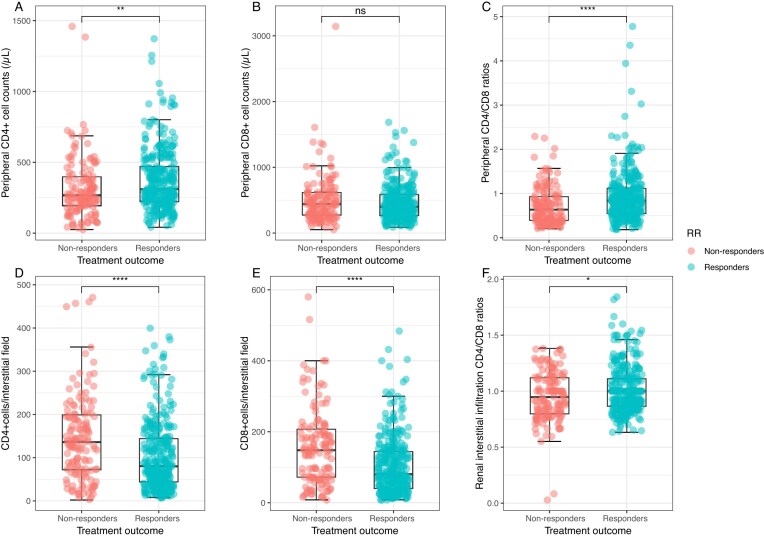
Distribution of circulating CD4^+^ lymphocytes (A), circulating CD8^+^ lymphocytes (B), circulating CD4/CD8 ratios (C), renal interstitial CD4^+^ lymphocytes (D), renal interstitial CD8^+^ lymphocytes (E), and renal interstitial CD4/CD8 ratios (F) stratified by treatment response.*P < .05, **P < .01, ***P < .001, ****P < .0001. Boxplot: boxplot medians (center lines), interquartile ranges (box ranges), whisker ranges.

The circulating T lymphocytes, particularly CD4 and the CD4/CD8 ratios, were significantly higher in responders than in nonresponders. However, nonresponders had more infiltrated CD4 and CD8 counts in the tubulointerstitium, though responders had higher CD4/CD8 ratios (Fig. 2).

Multivariable-adjusted analyses were performed to evaluate the prognostic significance of circulating and renal interstitial T lymphocyte metrics in predicting therapeutic response. Elevated peripheral CD4^+^ T-cell counts were consistently associated with improved treatment outcomes across all models (*P* < .05). Moreover, the peripheral CD4/CD8 ratio exhibited a strong positive association with treatment response. This association remained significant both in multivariable-adjusted models and after Bonferroni correction (Adjusted *P*-value = .0058). Tertile analysis revealed a dose-dependent relationship. The odds ratio for treatment response in the highest vs. lowest tertile was 2.697 (95% CI: 1.474–4.935; *P* = .001) in Model II, as corroborated by significant trend tests (*P* = .001). A significant dose-dependent relationship was observed for the peripheral CD4/CD8 ratio, which remained significant after Bonferroni correction (*P* for trend <.0125). Conversely, CD8^+^ cell counts did not exhibit significant associations (*P* = .243). However, lower renal interstitial CD8^+^ T-cell counts were associated with improved treatment response across all models (*P* < .05). Renal interstitial CD4^+^ T-cell counts also showed a strong negative association with treatment response in univariable analysis (*P* < .001), although this association was attenuated and no longer statistically significant after adjustment for clinicopathological confounders (*P* = .154). Moreover, increased renal interstitial CD4/CD8 ratios were strongly linked to favorable outcomes, with effect sizes amplifying after adjustment (Model II: OR = 8.312; *P* = .00037). Nevertheless, tertile analysis of renal interstitial CD4/CD8 ratios did not reveal significant associations (Table 5).

**Table 5:** Associations of peripheral and renal T-cell profiles with response in lupus nephritis.

Exposure	Response rate (%)	Crude model		Model I		Model II		
		OR (95%CI)	*P*-value	OR (95%CI)	*P*-value	OR (95%CI)	*P*-value	Adj. *P*-value
***Peripheral CD4^+^cell counts***		1.001 (1.000, 1.002)	**.01029**	1.002 (1.001, 1.003)	**.00182**	1.002 (1.001, 1.003)	**.00399**	.01596
Peripheral **CD4^+^ cell counts tertile**								
**T1 (25.00–244.00)**	87/141 (61.70%)	1.0		1.0		1.0		
**T2 (245.00–395.00)**	94/140 (67.14%)	1.268 (0.777, 2.069)	.34122	1.221 (0.732, 2.036)	.44499	0.976 (0.557, 1.710)	.93357	1.000
**T3 (396.00–1459.00)**	109/143 (76.22%)	1.990 (1.191, 3.325)	**.00861**	2.160 (1.250, 3.732)	**.00578**	1.971 (1.083, 3.585)	**.02624**	.10496
***P* for trend**		**.00882**		**.00620**		**.02876**		.11504
***Peripheral CD8^+^cell counts***		1.000 (0.999, 1.000)	.24284	1.000 (0.999, 1.000)	.33375	1.000 (0.999, 1.000)	.22161	.88644
Peripheral **CD8^+^ cell counts tertile**								
**T1 (52.00–313.00)**	96/141 (68.09%)	1.0		1.0		1.0		
**T2 (315.00–518.00)**	99/140 (70.71%)	1.132 (0.681, 1.881)	.63261	1.145 (0.672, 1.949)	.61866	1.127 (0.633, 2.008)	.68468	1.000
**T3 (521.00–3142.00)**	95/143 (66.43%)	0.928 (0.565, 1.523)	.76684	0.992 (0.582, 1.691)	.97578	0.835 (0.459, 1.518)	.55411	1.000
***P* for trend**		.76250		.97780		.56536		1.000
***Peripheral CD4/CD8 ratios***		2.451 (1.485, 4.045)	**.00045**	2.626 (1.550, 4.447)	**.00033**	2.462 (1.414, 4.288)	**.00146**	**.00584^a^**
Peripheral **CD4/CD8 ratios tertile**								
**Lower (0.18–0.58)**	80/141 (56.74%)	1.0		1.0		1.0		
**Middle (0.59–0.93)**	101/141 (71.63%)	1.925 (1.174, 3.159)	**.00950**	1.904 (1.133, 3.200)	**.01498**	1.870 (1.061, 3.294)	**.03033**	.12132
**Upper (0.94–4.78)**	109/142 (76.76%)	2.519 (1.509, 4.205)	**.00041**	2.762 (1.604, 4.757)	**.00025**	2.697 (1.474, 4.935)	**.00129**	**.00516^a^**
***P* for trend**		**.00034**		**.00021**		**.00118**		**.00472^a^**
***CD4^+^cells/mm²***		0.995 (0.993, 0.997)	**.00003**	0.996 (0.993, 0.998)	**.00032**	0.998 (0.995, 1.001)	.15414	.61656
**Interstitial CD4^+^ cell counts tertile**								
**T1 (2.00–60.00)**	112/141 (79.43%)	1.0		1.0		1.0		
**T2 (64.00–136.00)**	98/137 (71.53%)	0.651 (0.375, 1.130)	.12684	0.619 (0.351, 1.091)	.09734	0.809 (0.429, 1.524)	.51227	1.000
**T3 (140.00–472.00)**	80/146 (54.79%)	0.314 (0.186, 0.529)	**.00001**	0.344 (0.199, 0.596)	**.00014**	0.511 (0.268, 0.975)	**.04170**	.16680
***P* for trend**		**<.00001**		**.00013**		**.03653**		.14612
***CD8^+^cells/mm²***		0.994 (0.992, 0.997)	<.00001	0.994 (0.992, 0.997)	<.00001	0.996 (0.993, 0.999)	.00542	.02168
**Interstitial CD8^+^ cell counts tertile**								
**T1 (8.00–60.00)**	106/130 (81.54%)	1.0		1.0		1.0		
**T2 (64.00–143.00)**	107/144 (74.31%)	0.655 (0.367, 1.169)	.15219	0.617 (0.340, 1.123)	.11404	0.756 (0.392, 1.458)	.40450	1.000
**T3 (144.00–580.00)**	77/150 (51.33%)	0.239 (0.138, 0.413)	**<.00001**	0.241 (0.136, 0.428)	**<.00001**	0.343 (0.177, 0.666)	**.00159**	**.00636^a^**
***P* for trend**		**<.00001**		**<.00001**		**.00092**		**.00368^a^**
***Renal interstitial CD4/CD8 ratios***		4.304 (1.571, 11.792)	**.00454**	6.614 (2.255, 19.398)	**.00058**	8.312 (2.593, 26.645)	**.00037**	**.00148^a^**
**Renal interstitial CD4/CD8 ratios tertile**								
**Lower (0.03–0.88)**	83/137 (60.58%)	1.0		1.0		1.0		
**Middle (0.89–1.03)**	109/145 (75.17%)	1.970 (1.184, 3.278)	**.00908**	2.138 (1.250, 3.658)	**.00554**	1.699 (0.939, 3.072)	.07956	.31824
**Upper (1.04–1.84)**	98/142 (69.01%)	1.449 (0.884, 2.375)	.14103	1.736 (1.026, 2.937)	**.03994**	1.713 (0.966, 3.039)	.06573	.26292
***P* for trend**		.1365		**.03852**		.06185		.24740

Crude model adjusts for: None;

Adjust I model adjust for: gender, age, SLEDAI, hypertension, LN duration;

Adjust II model adjust for: gender, age, SLEDAI, hypertension, LN duration, eGFR, UPRO, Anti-dsDNA, C3, ISN/RPS classification, AI, CI, acute renal tubular injury, IFTA, and therapy class.

Abbreviations: OR, odds ratio; CI, confidence interval; Ref, reference group.

a Bonferroni correction was applied for multiple testing within each exposure variable. The significance threshold for each variable was set at P < .0125

Bold values indicate statistically significant associations (P < 0.05). Bonferroni correction was applied for multiple testing within each exposure variable. The significance threshold for each variable was set at P < 0.0125. Crude model adjusts for: None; Model I adjusts for: gender, age, SLEDAI, hypertension, LN duration; Model II adjusts for: gender, age, SLEDAI, hypertension, LN duration, eGFR, UPRO, Anti-dsDNA, C3, ISN/RPS classification, AI, CI, acute renal tubular injury, IFTA, and therapy class. OR, odds ratio; CI, confidence interval.

### Incremental predictive value of the peripheral CD4/CD8 ratio

We further evaluated whether the peripheral CD4/CD8 ratio provided predictive value over established clinical and pathological prognostic indicators. When added to a baseline model comprising SLEDAI, eGFR, UPRO, AI, and CI, the ratio significantly improved the model’s discriminative ability, with the AUC increasing from 0.678 (95% CI: 0.619–0.737) to 0.711 (95% CI: 0.656–0.766) (*P* for difference = .022). The full results of this model comparison, including reclassification metrics, are provided in Supplementary Table S4.

### Consistency across disease subgroups

Subgroup analyses were conducted to evaluate the associations between the peripheral CD4/CD8 ratios and treatment response in LN (Supplementary Table S5). Notably, most of these analyses indicated no significant differences within the groups, except that the positive association between the peripheral CD4/CD8 ratios and treatment response was more pronounced among male participants.

We performed a sensitivity analysis to assess the consistency of the association between the peripheral CD4/CD8 ratio and treatment response across different immunosuppressive regimens. Although the point estimates of the odds ratios varied among subgroups, a formal test for interaction was not statistically significant (*P* for interaction = .1476). The association was consistently positive across all treatment groups (Supplementary Table S5).

### Mediating role of renal T-cell infiltration in peripheral immunity-outcome relationships

Supplementary Table S6 presents the associations between the peripheral CD4/CD8 ratios and renal interstitial inflammatory cell subsets, which were determined by generalized linear models. After controlling for all confounding variables, the peripheral CD4/CD8 ratios were found to be positively correlated with the interstitial CD4/CD8 ratios (β = 0.381, 95% CI = 0.134–0.628, *P* = .003) and negatively correlated with interstitial CD4^+^ cells (β = −0.001, 95% CI = −0.001 to −0.000, *P* = .041). In contrast to CD4^+^ cells, interstitial CD8^+^ cell counts showed a significant negative association with peripheral CD4/CD8 ratios in the fully adjusted model (β = −0.001, 95% CI = −0.001 to −0.000, *P* = .014).

**Figure 3:**
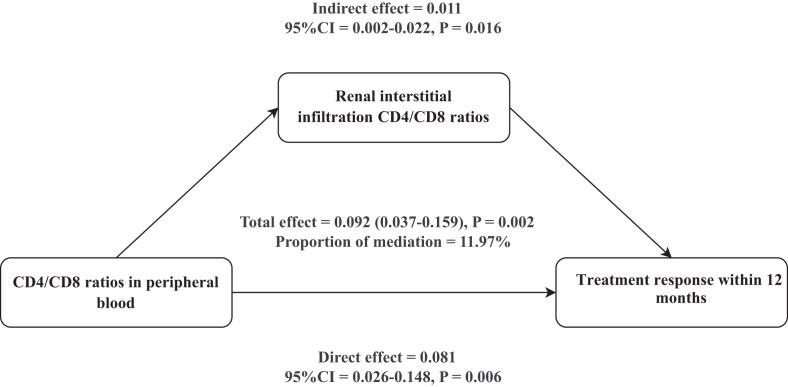
Renal interstitial CD4/CD8 ratios mediate the effect of peripheral CD4/CD8 ratios on treatment response.

A mediation analysis was conducted to assess whether the relationship between the peripheral CD4/CD8 ratios and 12-month renal remission was mediated by the renal interstitial CD4/CD8 ratios. All models were adjusted for variables such as gender, age, SLEDAI, hypertension, LN duration, eGFR, UPRO, Anti-dsDNA, C3, ISN/RPS classification, AI, CI, acute renal tubular injury, IFTA, and therapy class. The findings indicate that the peripheral CD4/CD8 ratios influence treatment response through both direct effects and renal interstitial-mediated effects, which accounted for 11.97% of the association (Supplementary Table S7 and Fig. 3).

### Association between peripheral CD4/CD8 ratio and long-term renal survival

**Figure 4:**
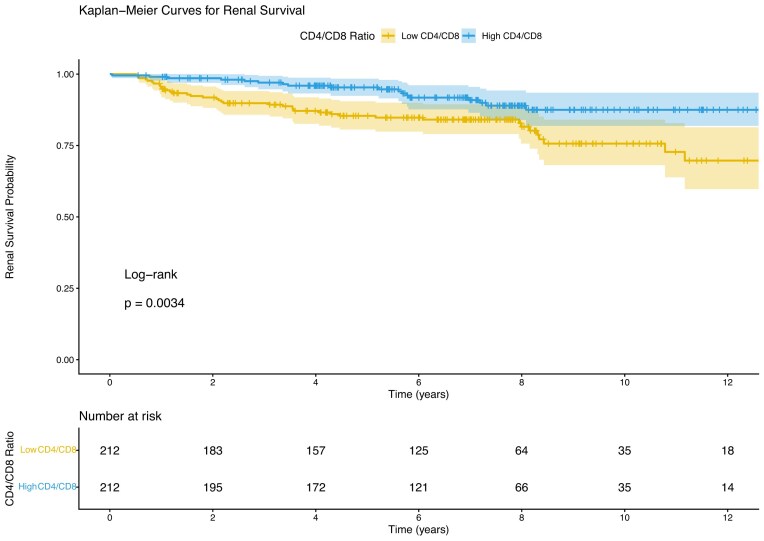
Kaplan-Meier curve for renal survival stratified by peripheral CD4/CD8 ratio.

During a median follow-up of 6.91 years (IQR, 5.12–8.54 years), 57 patients (13.4%) reached the composite renal endpoint (doubling of SCr or ESKD). Kaplan-Meier analysis revealed that patients with a baseline peripheral CD4/CD8 ratio above the median had significantly better renal survival compared to those with a ratio below the median (log-rank *P* = .0034; Fig. 4). In Cox regression analysis, a higher peripheral CD4/CD8 ratio was associated with a lower hazard of the composite endpoint in univariable analysis (HR = 0.44, 95% CI: 0.25–0.78, *P* = .0043) and after adjustment for demographic and clinical activity variables (Model I: HR = 0.47, 95% CI: 0.27–0.83, *P* = .0090) (Supplementary Table S8). However, after further adjustment for baseline renal function, proteinuria, CI, and treatment regimen (Model II), the association was attenuated and no longer statistically significant (HR = 0.68, 95% CI: 0.34–1.35, *P* = .273) (Supplementary Table S8).

## DISCUSSION

Our study found that in LN patients, those who responded to treatment had higher circulating CD4 levels and CD4/CD8 ratios, while nonresponders showed more T lymphocyte infiltration in the renal tubulointerstitium. The association between peripheral and renal interstitial T lymphocyte profiles and treatment response remained significant after adjustments. Importantly, our study demonstrates that the peripheral CD4/CD8 ratio offers incremental predictive value for treatment response over routinely collected clinical and histologic parameters, as evidenced by a significant improvement in model discrimination. Mediation analysis revealed that renal interstitial T-cell infiltration may be one pathway linking peripheral immunity to clinical outcomes.

Wang *et al*. found that compared with patients with membranous nephropathy, those with LN exhibited a significantly reduced proportion of CD4^+^ T cells and an increased proportion of CD8^+^ T cells in peripheral blood, primarily attributed to a decline in CD4^+^ cell numbers [9]. This finding aligns with the results of the current study, which demonstrated a marked reduction in peripheral CD4^+^ T cells relative to CD8^+^ T cells. CD4^+^ T cells have been shown to secrete key pro-inflammatory mediators, including interferon-γ (IFN-γ), interleukin-2 (IL-2), and related cytokines that facilitate early inflammatory responses. The observed decrease in peripheral CD4^+^ T cells may be attributed to their recruitment and migration to inflamed tissue in target organs [14]. In contrast, CD8^+^ T cells from the peripheral blood of patients with SLE often exhibit diminished effector functions, such as reduced production of granzyme B and perforin, rather than a numerical decrease [15]. This impaired cytolytic function in CD8^+^ T cells is likely a contributing factor to the pathogenesis of autoimmunity [16]. Studies involving lupus-prone mice with genetically ablated perforin production have demonstrated accelerated disease progression, thereby underscoring the critical role of cytotoxic T lymphocytes (CTLs) in mitigating autoimmunity [17]. These findings suggest the role of CD8^+^ T cells in the establishment and maintenance of peripheral tolerance, with defects in their cytolytic function unable to remove autoreactive B cells, ultimately propagating autoimmune pathogenesis.

The association between a lower peripheral CD4/CD8 ratio and poor treatment response prompts inquiry into its underlying immunologic drivers. Recent evidence suggests that lymphopenia in SLE may reflect enhanced interferon (IFN) activity. The type I IFN signature, a hallmark of SLE, has been associated with reduced naïve CD4^+^ T cell populations, as demonstrated in single-cell studies of SLE patients [18]. Furthermore, lymphopenia itself is considered a reflection of IFN pathway activation in SLE [19]. In our study, the reduced peripheral CD4/CD8 ratio observed in treatment nonresponders may therefore reflect heightened IFN activity, which could contribute to both global disease activity and treatment resistance in LN. This IFN-driven immunopathology might promote renal injury through multiple mechanisms, including enhanced antigen presentation, B-cell activation, and tissue inflammation. Our findings align with this paradigm, suggesting that peripheral T-cell profiling could serve as an accessible biomarker for IFN hyperactivity and help identify patients who might benefit from targeted anti-IFN therapies. Furthermore, this IFN-rich systemic milieu may critically influence the nature and intensity of T-cell infiltration into the renal interstitium, the prognostic impact of which is explored below.

Consequently, peripheral blood T-lymphocyte subsets in SLE patients exhibit significant abnormalities and dynamic alterations. Notably, the peripheral blood CD4/CD8 ratio in particular demonstrates a disease-specific biomarker, correlating with disease activity. This ratio serves as a critical indicator of immune system health and is associated with the prognosis of various diseases. For instance, in individuals infected with HIV, a low CD4/CD8 ratio is typically indicative of immune senescence and is correlated with an increased risk of cancer and mortality. Research has demonstrated that the CD4/CD8 ratio is intricately linked to the activation and proliferation capacity of T cells, with lower ratios being associated with mitochondrial dysfunction and elevated levels of inflammatory markers [20]. Numerous studies have identified the CD4/CD8 ratio as a pivotal marker of immune imbalance in autoimmune diseases.

While our study elucidates the strong association between the peripheral CD4/CD8 ratios and therapeutic response in LN, the underlying mechanisms driving these associations remain unclear. We posit that the aberrant infiltration of T lymphocyte subsets within the renal interstitium contributes to the pathogenesis of LN and consequently modulates therapeutic outcomes, potentially elucidating the positive association between the peripheral CD4/CD8 ratios and therapeutic response.

The subtypes of infiltrating T lymphocytes in renal tissue vary across different kidney diseases, resulting in distinct effects. In the context of diabetic nephropathy, increased infiltration of CD4^+^ T cells in the renal interstitium is associated with a more pronounced decline in renal function and more severe renal pathology, and CD4^+^ T cell infiltration is significantly correlated with adverse renal outcomes in these patients [21]. In ANCA-associated nephropathy, the renal interstitial infiltration is predominantly composed of T cells, in contrast to sparse B cell involvement. However, the density of T cell infiltration suggests no correlation between T cell density and baseline renal function, nor with therapeutic response or long-term prognosis [22]. In anti-glomerular basement membrane (anti-GBM) nephropathy, CD4^+^ T cell infiltration is more prominent around cellular crescents, and its density is significantly positively correlated with SCr levels at diagnosis and accelerates renal function deterioration, while CD8^+^ T cells predominantly localize in regions of Bowman’s capsule rupture, a process mediated by the release of perforin and granzyme-B that lyses parietal epithelial cells. The infiltration of CD3^+^ T cells in the peri-glomerular formed by crescents is significantly higher than that in the glomeruli with only mild mesangial proliferation [23]. However, in LN, earlier studies showed that renal infiltrating CD8^+^ T cells were the predominant infiltrating cells in patients with type III or IV LN [11], and the number of CD8^+^ T cell infiltrates in renal tissue positively correlates with podocyte loss. CD8^+^ T cells directly contact podocytes and induce apoptosis, thereby leading to proteinuria and deterioration of renal function [24]. Previous studies in our center have also shown that renal interstitial CD8^+^T cell infiltration is closely related to the degree of clinicopathological injury in LN patients [25]. In this study, we found that renal interstitial infiltration of CD8^+^T cells was strongly associated with treatment response, even after correction for clinicopathological parameters. We also found that the CD4/CD8 ratios of renal interstitial infiltration were strongly associated with treatment response, even after adjustment for clinicopathological parameters.

In the present study, we hypothesized that renal interstitial CD4/CD8 ratios would have significant mediating effects in the association of peripheral CD4/CD8 ratios with therapeutic effects in LN patients. Analyses demonstrated that renal interstitial CD4/CD8 ratios accounted for 11.97% of this mediation effect. The modest mediation proportion suggests the renal interstitial ratio is one of several pathways. The majority of the effect is likely direct or mediated through other unmeasured pathways, such as the role of peripheral T-cells in humoral immunity, secretion of soluble cytokines, or functional T-cell polarization beyond the CD4/CD8 classification.

Furthermore, our extended survival analysis demonstrated that a lower peripheral CD4/CD8 ratio was significantly associated with an increased risk of long-term adverse renal outcomes (creatinine doubling/ESKD) over a median follow-up of nearly 7 years. While this association was attenuated upon full adjustment for established clinical and histopathological risk factors, the univariable and partially adjusted associations suggest that peripheral T-cell imbalance may signify a more aggressive disease phenotype with poorer long-term prognosis.

However, this study has several limitations. It was a retrospective, single-center study that focused solely on baseline peripheral and renal interstitial T lymphocyte profiles, limiting the availability of follow-up data. We did not examine the relationship between therapy, particularly glucocorticoids, and T cell subpopulations. Furthermore, the absence of a control cohort with other forms of glomerulonephritis limits our ability to determine whether the observed T-cell profiles are specific to LN or represent a general response to immune-mediated kidney injury. Peripheral CD4/CD8 ratios can be influenced by factors not systematically adjusted for in our study, such as concurrent infections (e.g. CMV) or age-related immunosenescence. Our cohort consisted predominantly of young Chinese women with proliferative LN, which may limit the generalizability of our findings to other populations and requires external validation. This study utilized a historic cohort from 2010 to 2017. Treatment practices for LN continue to evolve, with increasing use of combination therapies and the introduction of new biologic agents. In our cohort, only three patients were treated with rituximab. Our findings have not been applied to LN patients receiving biologics and should be validated in recent cohorts. Future research should incorporate longitudinal data to better track changes in T lymphocyte levels and function over time. Moreover, potential limitations in the immunostaining method, such as antibody specificity, tissue fixation, and tissue quality, should be considered.

In conclusion, we have shown that peripheral and renal interstitial T lymphocytes are closely associated with treatment response, especially the CD4/CD8 ratios, both peripheral and renal interstitial. Crucially, mediation analysis revealed that the renal interstitial CD4/CD8 ratios significantly mediate the association between peripheral CD4/CD8 ratios and therapeutic outcomes, implicating T-cell redistribution as a mechanistic link in treatment response.

## Supplementary Material

sfag106_Supplemental_File

## Data Availability

The data underlying this article will be shared on reasonable request to the corresponding author.
