# Supplementary material for: Peripheral and renal interstitial T-cell profiles associated with treatment response in lupus nephritis: a retrospective cohort study
Source: Clin Kidney J. 2026 Mar 26;19(5):sfag106. doi: 10.1093/ckj/sfag106 (PMC13133625; doi:10.1093/ckj/sfag106)
Supplement: sfag106_Supplemental_File [file sfag106_supplemental_file.docx]

Table S1. Scoring System and Cohort Breakdown of the NIH Activity and Chronicity Indices

| **Index / Component** | **Score Range** | **Total**  **(N=424)** | **Non-responders**  **(n=134)** | **Responders**  **(n=290)** | **P-value** |
| --- | --- | --- | --- | --- | --- |
| **Activity Index (AI)** | 0-24 | 6.50 (4.00-9.00) | 6.00 (4.00-9.00) | 7.00 (4.00-9.00) | 0.935 |
| **Glomerular hypercellularity** | 0-3 | 1.00 (1.00-2.00) | 1.00 (1.00-2.00) | 1.00 (1.00-2.00) | 0.782 |
| **Glomerular neutrophils/karyorrhexis** | 0-3 | 1.00 (0.00-1.00) | 1.00 (0.00-1.00) | 1.00 (0.00-1.00) | 0.654 |
| **Fibrinoid necrosis** | 0-3 | 0.00 (0.00-1.00) | 0.00 (0.00-1.00) | 0.00 (0.00-1.00) | 0.423 |
| **Hyaline deposits** | 0-3 | 1.00 (0.00-1.00) | 1.00 (0.00-1.00) | 1.00 (0.00-1.00) | 0.215 |
| **Cellular/fibrocellular crescents** | 0-3 | 1.00 (0.00-2.00) | 1.00 (0.00-2.00) | 1.00 (0.00-2.00) | 0.089 |
| **Interstitial inflammation** | 0-3 | 1.00 (1.00-1.00) | 1.00 (1.00-1.00) | 1.00 (1.00-1.00) | 0.327 |
| **Chronicity Index (CI)** | 0-12 | 2.00 (1.00-3.00) | 2.00 (1.00-4.00) | 1.00 (0.00-3.00) | <0.001 |
| **Glomerular sclerosis** | 0-3 | 1.00 (0.00-1.00) | 1.00 (0.00-2.00) | 0.00 (0.00-1.00) | <0.001 |
| **Fibrous crescents** | 0-3 | 0.00 (0.00-0.00) | 0.00 (0.00-0.00) | 0.00 (0.00-0.00) | 0.134 |
| **Tubular atrophy** | 0-3 | 1.00 (0.00-1.00) | 1.00 (1.00-1.00) | 1.00 (0.00-1.00) | 0.008 |
| **Interstitial fibrosis** | 0-3 | 1.00 (0.00-1.00) | 1.00 (1.00-1.00) | 1.00 (0.00-1.00) | 0.003 |

Table S2. Multinomial Logistic Regression Analysis of the Association Between Peripheral CD4/CD8 Ratio and Different Grades of Treatment Response (Complete Remission, Partial Remission, and No Remission)

| **Outcome Comparison** | **OR (95% CI)** | **P-value** | **Adjusted P-value** |
| --- | --- | --- | --- |
| **PR vs. NR** | 2.74 (1.47, 5.12) | 0.002 | 0.003* |
| **CR vs. NR** | 2.37 (1.32, 4.26) | 0.004 | 0.008* |

*P-values are adjusted for multiple comparisons using the Bonferroni method (significance threshold: P < 0.025).

Table S3. Correlations Between Peripheral and Renal Interstitial T-Cell Subset Densities

| **Comparison** | **Rho** | **P-value** | **Adjusted P-value** |
| --- | --- | --- | --- |
| **Peripheral vs. Renal Interstitial CD4⁺ cells** | -0.120 | 0.014 | 0.027 |
| **Peripheral vs. Renal Interstitial CD8⁺ cells** | -0.041 | 0.399 | 0.799 |

*P-values are adjusted for multiple comparisons using the Bonferroni method (significance threshold: P < 0.025).

Table S4.Comparison of Prediction Models for Treatment Response with and without the Peripheral CD4/CD8 Ratio

|  | **Model 1** | **Model 2** | **P Value for Difference** |
| --- | --- | --- | --- |
| **Discrimination** |  |  |  |
| **AUC (95% CI)** | 0.678 (0.619 - 0.737) | 0.711 (0.656 - 0.766) | 0.022 |
| **Calibration** |  |  |  |
| **Best Threshold** | 0.527 | 0.732 | - |
| **Classification at Best Threshold** |  |  |  |
| **Sensitivity** | 0.797 | 0.714 | - |
| **Specificity** | 0.560 | 0.627 | - |
| **Accuracy** | 0.722 | 0.686 | - |
| **Positive Predictive Value** | 0.797 | 0.805 | - |
| **Reclassification** |  |  |  |
| **Net Reclassification Index (NRI)** |  |  |  |
| **Overall NRI (95% CI)** | Reference | -0.016 (-0.086 to 0.055) | 0.663 |
| **NRI for Events (95% CI)** | Reference | -0.083 (-0.126 to -0.040) | <0.001 |
| **NRI for Non-Events (95% CI)** | Reference | 0.067 (0.012 to 0.123) | 0.018 |
| **Integrated Discrimination Improvement (IDI)** |  |  |  |
| **IDI (95% CI)** | Reference | -0.016 (-0.086 to 0.055) | 0.664 |

Model 1: logit(RR) = -1.23723 +0.06173*SLEDAI +0.01506*eGFR +0.00136*UPRO +0.02283*AI -0.12600*CI

Model 2: logit(RR) = -2.13518 +0.06893*SLEDAI +0.01518*eGFR +0.00456*UPRO +0.02340*AI -0.10864*CI +0.87602*peripheral CD4/CD8 ratio

Table S5. Stratified Associations Between Peripheral CD4/CD8 Ratios and Responders Across Lupus Nephritis Disease Classes

| **Variables** | **OR (95% CI)** | **P-value** | **P for interaction** |
| --- | --- | --- | --- |
| **Gender** |  |  | **0.0157** |
| **Female** | 1.890 (1.114, 3.206) | 0.0183 |  |
| **Male** | 10.887 (2.548, 46.519) | 0.0013 |  |
| **Age (years)** |  |  | 0.1343 |
| **≤30** | 3.690 (1.725, 7.890) | 0.0008 |  |
| **>30** | 1.716 (0.895, 3.291) | 0.1038 |  |
| **SLEDAI (2K)** |  |  | 0.9873 |
| **≤14** | 2.528 (1.356, 4.716) | 0.0035 |  |
| **>14** | 2.550 (1.075, 6.052) | 0.0337 |  |
| **LN duration (months)** |  |  | 0.0984 |
| **≤2** | 1.580 (0.816, 3.058) | 0.1745 |  |
| **>2** | 3.649 (1.742, 7.641) | 0.0006 |  |
| **Immunosuppressive therapy-naive, n (%)** |  |  | 0.7922 |
| **YES** | 2.019 (0.746, 5.459) | 0.1665 |  |
| **NO** | 2.358 (1.326, 4.194) | 0.0035 |  |
| **Hb (g/L)** |  |  | 0.7997 |
| **≤110** | 2.547 (1.377, 4.715) | 0.0029 |  |
| **>110** | 2.220 (0.941, 5.241) | 0.0687 |  |
| **eGFR(mL/min per 1.73 m2)** |  |  | 0.7904 |
| **≤90** | 2.318 (1.197, 4.490) | 0.0127 |  |
| **>90** | 2.667 (1.200, 5.929) | 0.0161 |  |
| **UPRO (g/24h)** |  |  | 0.8782 |
| **≤3.5** | 2.552 (1.247, 5.224) | 0.0104 |  |
| **>3.5** | 2.360 (1.174, 4.743) | 0.0159 |  |
| **ISN/RPS classification, n (%)** |  |  | 0.8911 |
| **Class II** | 1.948 (0.086, 44.130) | 0.6753 |  |
| **Class III** | 1.522 (0.395, 5.856) | 0.5415 |  |
| **Class III+V** | 1.677 (0.486, 5.789) | 0.4133 |  |
| **Class IV** | 2.357 (1.016, 5.470) | 0.0459 |  |
| **Class IV+V** | 2.927 (1.055, 8.124) | 0.0392 |  |
| **Class V** | 4.599 (1.083, 19.524) | 0.0386 |  |
| **Activity index** |  |  | 0.5502 |
| **≤6** | 2.165 (1.147, 4.086) | 0.0172 |  |
| **>6** | 2.947 (1.339, 6.486) | 0.0073 |  |
| **Chronicity index** |  |  | 0.7182 |
| **≤2** | 2.180 (1.139, 4.174) | 0.0187 |  |
| **>2** | 2.625 (1.213, 5.677) | 0.0142 |  |
| **Treatment** |  |  | 0.1476 |
| **Pred+MMF+CNIs** | 34.52 (3.14, 379.75) | 0.0038 |  |
| **Pred+CYC** | 1.03 (0.18, 5.89) | 0.9713 |  |
| **Pred+MMF** | 1.98 (0.16, 23.93) | 0.5905 |  |
| **Pred+CNIs** | 5.97 (0.40, 89.67) | 0.1959 |  |
| **Others** | 13.28 (0.55, 319.37) | 0.1109 |  |

SLEDAI, Systemic Lupus Erythematosus Disease Activity; LN, lupus nephritis; Hb, hemoglobin; eGFR, the estimated glomerular filtration rate; UPRO, urinary protein quantitation.

Table S6. Associations Between Peripheral CD4/CD8 Ratios and Renal Interstitial T-Cell Infiltration Density

|  | **β value** | **95%CI** | **P value** |
| --- | --- | --- | --- |
| **Renal interstitial CD4+cells** |  |  |  |
| **Crude model** | -0.001 | -0.001, 0.000 | 0.09539 |
| **Model I** | -0.001 | -0.001, -0.000 | **0.04172** |
| **Model II** | -0.000 | -0.001, 0.000 | 0.20387 |
| **Renal interstitial CD8+cells** |  |  |  |
| **Crude model** | -0.001 | -0.001, -0.000 | **0.00554** |
| **Model I** | -0.001 | -0.001, -0.000 | **0.00160** |
| **Model II** | -0.001 | -0.001, -0.000 | **0.01395** |
| **Renal interstitial CD4/8 ratios** |  |  |  |
| **Crude model** | 0.388 | 0.146, 0.629 | **0.00178** |
| **Model I** | 0.381 | 0.139, 0.623 | **0.00214** |
| **Model II** | 0.381 | 0.134, 0.628 | **0.00262** |

Crude model adjusts for: None;

Adjust I model adjust for: gender, age, SLEDAI, hypertension, LN duration;

Adjust II model adjust for: gender, age, SLEDAI, hypertension, LN duration, eGFR, UPRO, Anti-dsDNA, C3, ISN/RPS classification, AI, CI, acute renal tubular injury, IFTA, and therapy class.

Table S7. Analysis of the mediation by renal interstitial inflammatory cell profiles of the associations of peripheral CD4/CD8 ratios with treatment response

|  | **β (95% CI), P value** | | |  |
| --- | --- | --- | --- | --- |
|  | Total effect | Indirect effect | Direct effect | Mediation |
| **Renal interstitial CD4+cells** | 0.093209  (0.036065, 0.160632) **0.002** | 0.001807  (-0.001566, 0.007437) 0.350 | 0.091402  (0.035448, 0.160270) **0.002** | 1.94% |
| **Renal interstitial CD8+cells** | 0.092055  (0.036153,0.158337) **0.002** | 0.006791  (0.000126, 0.015478) **0.040** | 0.085264  (0.029529,0.153342) **0.004** | 7.38% |
| **Renal interstitial CD4/8 ratios** | 0.091945 (0.037313-0.158802) **0.002** | 0.011003 (0.002386-0.022159) **0.016** | 0.080942 (0.026475-0.147668) **0.006** | 11.97% |

Adjust for: gender, age, SLEDAI, hypertension, LN duration, eGFR, UPRO, Anti-dsDNA, C3, ISN/RPS classification, AI, CI, acute renal tubular injury, IFTA, and therapy class.

Table S8: Results of Cox proportional hazards models for the association between peripheral CD4/CD8 ratio and the composite renal endpoint.

| **Exposure** | **Crude model** | | **Model I** | | **Model II** | |
| --- | --- | --- | --- | --- | --- | --- |
|  | **OR (95%CI)** | **P-value** | **OR (95%CI)** | **P-value** | **OR (95%CI)** | **P-value** |
| **Peripheral CD4/CD8 ratios** | 0.39 (0.19, 0.80) | 0.0101 | 0.43 (0.21, 0.86) | 0.0181 | 0.69 (0.33, 1.48) | 0.3441 |
| **Peripheral CD4/CD8 ratios dichotomous** |  |  |  |  |  |  |
| **Low** | 1.0 |  | 1.0 |  | 1.0 |  |
| **High** | 0.44 (0.25, 0.78) | 0.0043 | 0.47 (0.27, 0.83) | 0.0090 | 0.68 (0.34, 1.35) | 0.2730 |

Crude model adjusts for: None;

Adjust I model adjust for: gender, age, SLEDAI, hypertension, LN duration;

Adjust II model adjust for: gender, age, SLEDAI, hypertension, LN duration, eGFR, UPRO, Anti-dsDNA, C3, ISN/RPS classification, AI, CI, acute renal tubular injury, IFTA, and therapy class.
